# The Overlap of Maternal and Neonatal Critical Care Admission in the United States: Trends and Risk Factors in 2016–2024

**DOI:** 10.3390/jcm15166357

**Published:** 2026-08-18

**Authors:** Tariq Al Bahhawi

**Affiliations:** Family and Community Medicine Department, College of Medicine, Jazan University, Jazan 45142, Saudi Arabia; tbahhawi@jazanu.edu.sa

**Keywords:** maternal intensive care, neonatal intensive care unit, concurrent ICU admission, perinatal morbidity

## Abstract

**Background/Objectives:** Maternal and neonatal intensive care unit (ICU) admissions are markers of severe perinatal morbidity, yet their co-occurrence within the same birth remains poorly characterized at the population level. The objective of this study was to examine temporal trends, distribution, and maternal risk factors associated with concurrent maternal and neonatal ICU admission among singleton births in the United States. **Methods:** This population-based, serial cross-sectional study used the US Centers for Disease Control and Prevention Wide-Ranging Online Data for Epidemiologic Research (WONDER) database from 2016 to 2024. Singleton live births were included and classified into four mutually exclusive ICU phenotypes: maternal ICU admission only, neonatal ICU admission only, concurrent maternal–neonatal ICU admission, and neither. Annual rates per 1000 births were calculated, and temporal trends were assessed using joinpoint regression. Maternal characteristics associated with concurrent ICU admission were evaluated using descriptive analyses and crude relative risks. **Results:** The analytic cohort included 32,341,764 singleton births. Between 2016 and 2024, rates of neonatal ICU admission increased from 76.35 to 88.46 per 1000 births (average annual percent change [AAPC], 1.65%; 95% CI, 1.35–1.92%) and maternal ICU admission from 1.49 to 1.82 per 1000 births (AAPC, 2.48%; 95% CI, 1.73–3.14%). Concurrent maternal–neonatal ICU admission remained uncommon but increased from 0.70 to 0.92 per 1000 births (AAPC, 3.32%; 95% CI, 1.67–4.88%). Across gestational age, neonatal ICU admission without maternal ICU admission was the dominant phenotype, particularly at earlier gestational ages. Concurrent ICU admission showed a similar but attenuated pattern, whereas maternal ICU admission without neonatal ICU admission remained uncommon, with relatively higher rates at the earliest gestational ages. Concurrent ICU admission was associated with markers of maternal and pregnancy risk. The highest risks were observed among pregnancies complicated by eclampsia, pre-pregnancy diabetes, and pre-pregnancy hypertension, as well as among women with no prenatal care. Risk increased with advancing maternal age and higher body mass index, and disparities were observed across racial groups. **Conclusions:** Concurrent maternal and neonatal ICU admission is a rare but increasing outcome concentrated among high-risk pregnancies. These findings highlight the need for integrated maternal–neonatal risk assessment and continued surveillance of severe perinatal outcomes.

## 1. Introduction

Admission to an intensive care unit (ICU) during pregnancy or the perinatal period is widely used as a marker of critical maternal or neonatal illness. It reflects conditions associated with high clinical complexity and substantial resource use. Maternal ICU admission most commonly occurs in the context of severe obstetric complications such as hypertensive disorders of pregnancy, obstetric hemorrhage, sepsis, and cardiovascular instability [1]. Neonatal ICU admission reflects serious neonatal morbidity, often related to prematurity or fetal compromise [2].

Maternal ICU admission and neonatal ICU admission are routinely captured as distinct indicators of severe perinatal morbidity. Evidence suggests that the burden of both outcomes has increased over time [3,4,5]. Over the same period, the prevalence of key maternal risk factors, including obesity, diabetes, and advanced maternal age, has increased [6,7]. These factors are consistently associated with both severe maternal complications and adverse neonatal outcomes. Together, they increase the likelihood of escalation beyond routine obstetric and neonatal care [8,9].

Despite this shared clinical context, maternal ICU admission and neonatal ICU admission are most often examined as separate outcomes in both clinical studies and surveillance reports [2,3,4,5]. This separation overlooks pregnancies in which both mother and infant require critical care. Such pregnancies may reflect shared maternal–fetal disease processes rather than two unrelated manifestations of illness. Maternal and fetal wellbeing are physiologically linked, and a single underlying process, such as a hypertensive disorder of pregnancy or placental dysfunction, can affect both at once [10]. The same process may precipitate maternal critical illness while also causing fetal compromise or preterm birth requiring neonatal intensive care.

Consistent with this, concurrent maternal–neonatal ICU admission is uncommon, occurring in approximately 1.2 per 1000 live births in a Canadian population-based cohort, yet more than half of mothers admitted to an ICU had an infant admitted to a neonatal ICU [10]. Compared with maternal or neonatal ICU admission alone, concurrent admission carried higher maternal and neonatal mortality, longer hospital stay, and greater mother–infant separation through interfacility transfer [10]. Disparities are also more pronounced for this outcome, with differences in risk by maternal region of birth greater for concurrent admission than for maternal ICU admission alone [11]. These findings suggest that concurrent admission identifies a distinct clinical phenotype rather than the overlap of two unrelated processes. However, population-level trends and epidemiological patterns of concurrent maternal–neonatal ICU admission remain poorly characterized, particularly in the United States.

Using US national birth certificate data accessed through Centers for Disease Control and Prevention Wide-ranging Online Data for Epidemiologic Research (CDC WONDER) system, this study examines temporal trends in maternal, neonatal, and concurrent maternal–neonatal ICU admission among singleton births from 2016 to 2024, and describes the maternal demographic and clinical characteristics associated with concurrent admission.

## 2. Materials and Methods

### 2.1. Data Source and Study Population

This population-based serial cross-sectional analysis used publicly available aggregate natality data obtained from CDC WONDER [12]. CDC WONDER natality data are derived from U.S. standard birth certificates and are compiled annually by the National Center for Health Statistics, providing near-complete national coverage of all registered live births in the United States.

Data were extracted for the years 2016–2024. The study period began in 2016 because national birth certificate data on maternal and neonatal ICU admission are available or all reporting areas from this year onward. The study population consisted of singleton live births occurring in the United States during the study period. Analyses were restricted to singleton births to minimize the potential for double-counting of maternal ICU admission when neonatal outcomes are tabulated.

### 2.2. Outcome Measures

Maternal ICU admission was defined using the CDC WONDER natality variable indicating whether the mother was admitted to an intensive care unit during the delivery hospitalization. Neonatal ICU admission was defined using the corresponding variable indicating admission of the newborn to a neonatal intensive care unit. Both variables are derived from U.S. standard birth certificate items and are reported in CDC WONDER as binary indicators (yes or no). Records coded as unknown were excluded prior to tabulation.

Based on these indicators, births were classified into four mutually exclusive ICU phenotypes: maternal ICU admission without neonatal ICU admission, neonatal ICU admission without maternal ICU admission, concurrent maternal–neonatal ICU admission, and neither maternal nor neonatal ICU admission.

It should be noted that criteria for ICU admission are not standardized across hospitals in the United States, for either maternal or neonatal intensive care. No pregnancy-specific maternal ICU admission thresholds exist, and neonatal ICU admission practices vary considerably across institutions; for both, the decision to admit reflects institutional practice, unit capacity, and local referral patterns in addition to illness severity [13,14].

### 2.3. Covariates

Maternal characteristics available through CDC WONDER were used for stratified analyses of concurrent maternal–neonatal ICU admission. These included maternal age, maternal race, classified using the CDC WONDER single-race variable in six categories, timing of prenatal care initiation, Women, Infants, and Children (WIC) participation, pre-pregnancy body mass index category, pre-pregnancy diabetes, gestational diabetes, pre-pregnancy hypertension, gestational hypertension, eclampsia, tobacco use during pregnancy, and infections during pregnancy. All variables were defined using CDC WONDER standard recodes for the corresponding data years. Gestational age was defined using the obstetric estimate recode.

### 2.4. Statistical Analysis

For each calendar year, aggregate counts of births by ICU phenotype were extracted and rates per 1000 singleton live births were calculated using CDC WONDER–provided denominators. Confidence intervals (95%) for the annual rates were calculated using the Wilson score method for binomial proportions.

Temporal trends were evaluated using the Joinpoint Regression Program (version 5.4.0; National Cancer Institute, Bethesda, MD, USA) applied to annual rates [15,16]. Log-transformed rates were modeled assuming constant variance with first-order autocorrelation estimated from the data. Given nine annual observations, the maximum number of joinpoints was set to one, with a minimum of two observations from either end of the series. The final model was selected by permutation test (4499 permutations, significance level 0.05). Average annual percent change was estimated for the full period, with 95% confidence intervals obtained using the empirical quantile method with 5001 resamples. Relative risks were calculated as unadjusted rate ratios using aggregate counts and were not adjusted for potential confounders. Confidence intervals for stratum-specific rates were calculated using the normal approximation, and those for relative risks using the standard error of the log relative risk.

For maternal characteristics with more than 1% unavailable data, an additional missingness-pattern analysis was performed. Concurrent maternal–neonatal ICU admission rates were compared between births with recorded and unrecorded values for each characteristic. To evaluate whether record-level missingness reflected broader variation in birth certificate completeness, the proportion of births with an unknown maternal or neonatal ICU admission indicator was also calculated according to the availability of each characteristic. For eclampsia, record-level Unknown or Not Stated values were evaluated separately from Not Reported by jurisdiction, which represents reporting areas that did not collect the variable.

Data extraction, management, statistical analysis, and visualization were performed using R (R Foundation for Statistical Computing). All analyses adhered to CDC WONDER disclosure and suppression rules, and suppressed cells were not imputed or disaggregated. This study used publicly available, de-identified data and was exempt from institutional review board approval.

## 3. Results

Of 33,511,275 live births recorded between 2016 and 2024, 1,093,204 non-singleton births were excluded. After excluding births with unknown maternal ICU admission status (*n* = 52,903) and unknown neonatal ICU admission status (*n* = 23,404), the final analytic cohort comprised 32,341,764 singleton live births. In 2024, the cohort included 3,502,863 singleton births. Births were predominantly to women aged 25–34 years (57.9%), and most initiated prenatal care in the first trimester (74.1%). Pre-pregnancy obesity was present in 31.3% of pregnancies, whereas the prevalences of pre-pregnancy diabetes, pre-pregnancy hypertension, and tobacco use were 1.3%, 3.3%, and 2.4%, respectively (Table 1).

Rates of neonatal ICU admission increased from 76.35 (95% CI, 76.08–76.62) per 1000 births in 2016 to 88.46 (95% CI, 88.16–88.76) in 2024, while neonatal ICU admission without maternal ICU admission increased from 75.65 (95% CI, 75.38–75.91) to 87.54 (95% CI, 87.24–87.83) per 1000 births (Table 2). Maternal ICU admission increased from 1.49 (95% CI, 1.45–1.53) to 1.82 (95% CI, 1.78–1.87) per 1000 births, whereas maternal ICU admission without neonatal ICU admission increased from 0.79 (95% CI, 0.76–0.82) to 0.90 (95% CI, 0.87–0.93). Concurrent maternal–neonatal ICU admission remained uncommon but increased from 0.70 (95% CI, 0.68–0.73) to 0.92 (95% CI, 0.89–0.96) per 1000 births.

Joinpoint analysis showed significant upward trends for all outcomes (Table 3). Maternal ICU admission increased by an average of 2.48% annually (95% CI, 1.73–3.14; *p* < 0.001), compared with 1.66% (95% CI, 0.51–2.79; *p* = 0.005) for maternal ICU admission without neonatal ICU admission. The corresponding AAPCs for neonatal ICU admission and neonatal ICU admission without maternal ICU admission were similar, at 1.65% (95% CI, 1.35–1.92; *p* < 0.001) and 1.64% (95% CI, 1.38–1.88; *p* < 0.001), respectively. Concurrent maternal–neonatal ICU admission had the largest estimated AAPC, at 3.32% (95% CI, 1.67–4.88; *p* < 0.001).

**Figure 1:**
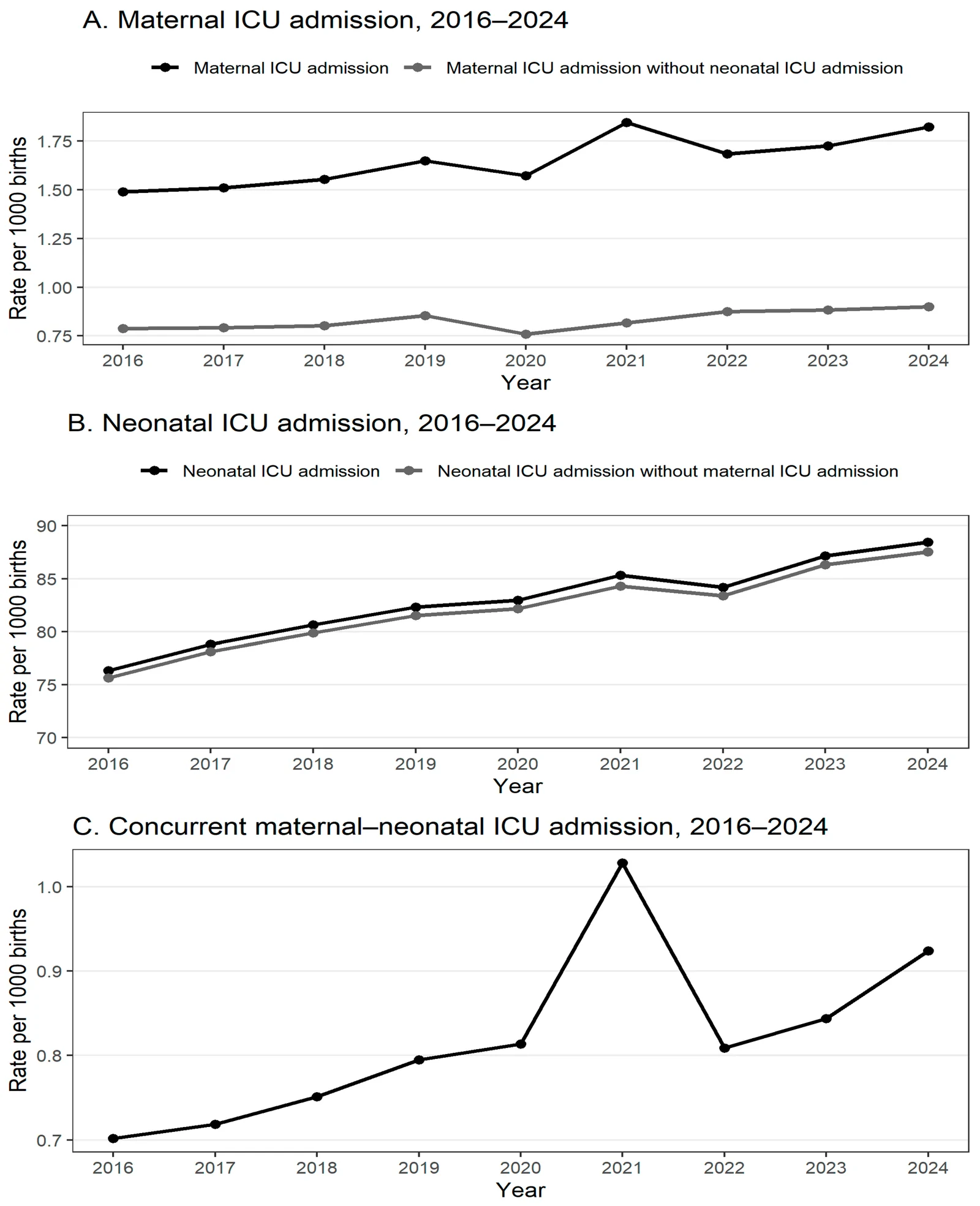
Temporal Trends in Maternal, Neonatal, and Concurrent Maternal–Neonatal Intensive Care Unit Admission Among Singleton Live Births in the United States, 2016–2024.

Figure 1 illustrates the temporal patterns of overall and non-overlapping maternal and neonatal ICU admissions and concurrent maternal–neonatal ICU admission. A transient increase occurred in 2021, most pronounced for concurrent maternal–neonatal ICU admission. Rates subsequently declined in 2022 but remained above their 2016 levels and increased again through 2024.

Relative risks (RR) are crude and are presented as descriptive risk stratification (Table 4). Marked differences in the risk of concurrent maternal–neonatal ICU admission were observed across maternal characteristics. The strongest associations were seen for severe maternal conditions, including eclampsia (RR, 23.15; 95% CI, 21.96–24.40), pre-pregnancy diabetes (RR, 6.57; 95% CI, 6.26–6.90), and pre-pregnancy hypertension (RR, 5.09; 95% CI, 4.90–5.29). Absence of prenatal care was also strongly associated with increased risk (RR, 6.11; 95% CI, 5.87–6.37).

Risk increased with maternal age, reaching an RR of 4.02 (95% CI, 3.49–4.64) among women aged 45 years or older, and with increasing pre-pregnancy body mass index, with an RR of 2.44 (95% CI, 2.34–2.55) in the highest category. Higher risks were also observed among Black or African American, American Indian or Alaska Native, and Native Hawaiian or other Pacific Islander mothers compared with White mothers. Tobacco use during pregnancy, infections during pregnancy, and WIC participation were also associated with increased risk.

Supplementary Table S1 reports the concurrent maternal–neonatal ICU admission rate and the proportion of births with an unknown maternal or neonatal ICU indicator, according to the availability of each characteristic with more than 1% unavailable data. Evaluation of missingness showed that record-level missingness was associated with both higher observed concurrent maternal–neonatal ICU admission rates and greater co-occurrence of unknown ICU indicators (Table S1). By contrast, births from jurisdictions that did not report eclampsia data had concurrent ICU admission rates and ICU-indicator completeness comparable with those of births with recorded eclampsia information.

**Figure 2:**
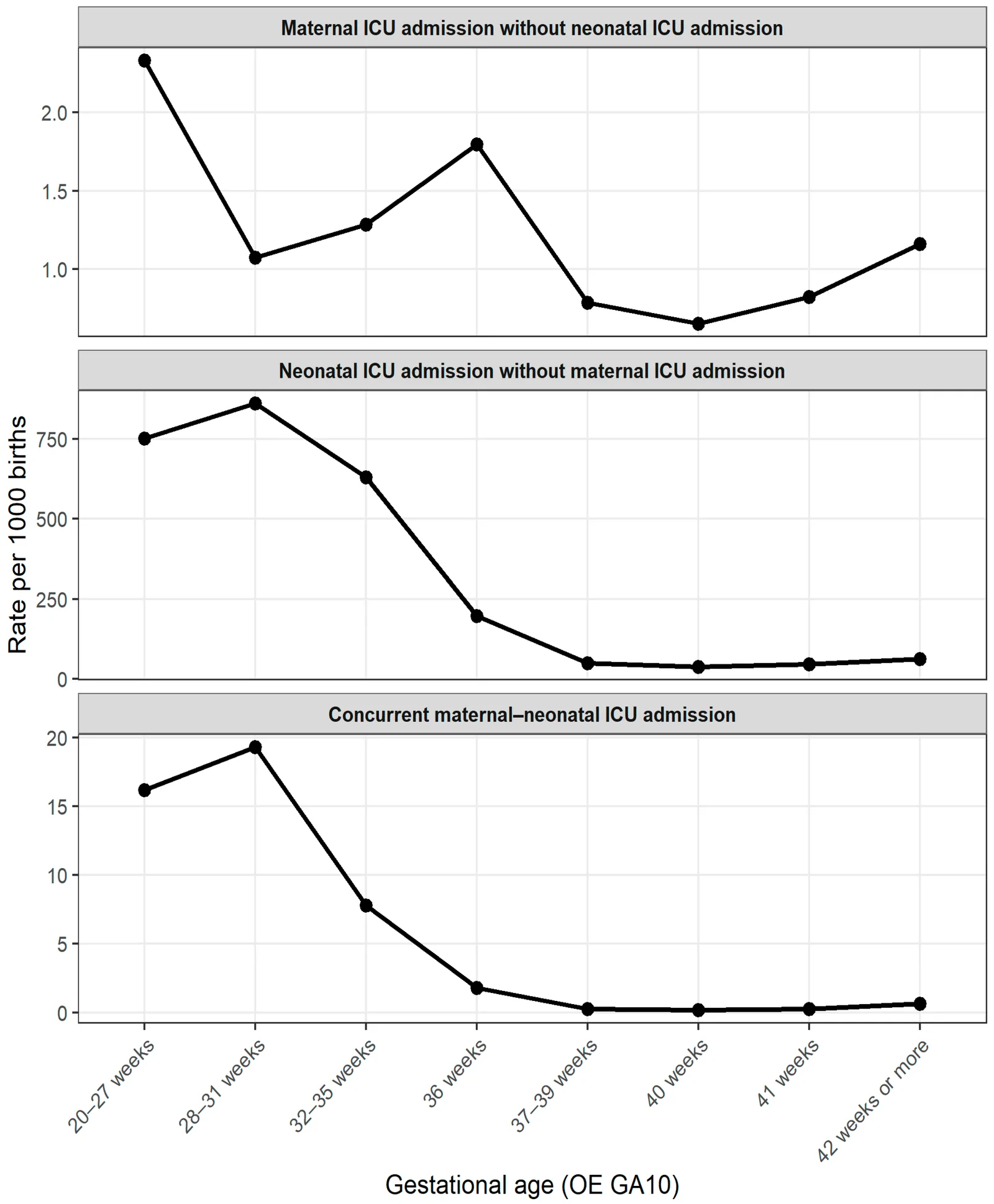
Distribution of Mutually Exclusive Maternal–Neonatal Intensive Care Unit Admission Phenotypes Across Gestational Age Among Singleton Live Births.

Rates of ICU phenotypes varied across gestational age (Figure 2). Neonatal ICU admission without maternal ICU admission was the dominant phenotype at earlier gestational ages and declined with increasing gestational age. Concurrent maternal–neonatal ICU admission followed a similar pattern, with higher rates at earlier gestational ages and lower rates at term. In contrast, maternal ICU admission without neonatal ICU admission remained uncommon but showed relatively higher rates at very early gestational ages, with more modest rates at 36 weeks and at post-term gestational ages.

## 4. Discussion

In this population-based study of more than 32 million singleton births in the United States between 2016 and 2024, both maternal and neonatal ICU admission rates increased over time. Neonatal ICU admission increased from 76.35 to 88.46 per 1000 births (AAPC, 1.65%), and maternal ICU admission from 1.49 to 1.82 per 1000 births (AAPC, 2.48%). Although concurrent maternal–neonatal ICU admission remained uncommon, it increased at a faster rate than either outcome alone (from 0.70 to 0.92 per 1000 births; AAPC, 3.32%). Concurrent ICU admission was more frequent among pregnancies complicated by severe maternal conditions, including eclampsia, pre-pregnancy diabetes, pre-pregnancy hypertension, among pregnancies without prenatal care, and at earlier gestational ages.

These findings are consistent with prior literature demonstrating increasing utilization of maternal and neonatal intensive care [3,4,5]. They further extend existing evidence by showing that the overlap of maternal and neonatal ICU admission represents an increasingly important component of perinatal morbidity at the population level. In a population-based study, concurrent maternal–neonatal ICU admission was associated with a substantially higher prevalence of maternal–infant interfacility separation, longer hospital stay, and increased mortality, reflecting the clinical severity of this phenotype [10]. Beyond acute outcomes, a recent cross-sectional study suggests that ICU admission is associated with increased postpartum psychological morbidity and impaired maternal–infant attachment, with the greatest burden observed when both mother and newborn require intensive care [17]. Together, these findings highlight concurrent ICU admission as a marker of both severe clinical and psychosocial vulnerability.

A transient increase in ICU admission rates was observed in 2021 across all phenotypes, with the largest proportional increase occurring in concurrent maternal–neonatal ICU admission. This pattern coincided with the COVID-19 pandemic in which severe maternal and neonatal morbidity were most frequently reported [18,19], and in which disease severity in pregnancy and health-system strain were greatest. However, joinpoint analysis did not identify a statistically significant change point, suggesting that this increase may represent a transient fluctuation rather than a distinct shift in the underlying trend. Beyond the direct effects of SARS-CoV-2 infection, pandemic-related disruptions to healthcare delivery, including strain on ICU capacity and critical care staffing shortages, may also have influenced ICU utilization independently of underlying illness severity [20]. Because ICU admission reflects healthcare organization as well as clinical need, the transient rise in 2021 may reflect both increased maternal and neonatal morbidity and pandemic-related changes in critical care delivery.

The markedly elevated risk of concurrent maternal–neonatal ICU admission among pregnancies complicated by eclampsia, pre-pregnancy diabetes, and pre-pregnancy hypertension reflects a clinically coherent pattern of shared maternal–fetal vulnerability [6,7,8,9]. Eclampsia showed the strongest association and illustrates this link most directly. Impaired trophoblast invasion and incomplete spiral artery remodeling produce placental ischemia and release of antiangiogenic factors, and the resulting endothelial dysfunction can affect multiple maternal organ systems and the fetus [10,21]. Maternal multisystem dysfunction may therefore develop alongside compromised fetal oxygenation and growth, frequently necessitating preterm delivery and neonatal intensive care [10,21]. Preeclampsia additionally increases the risk of peripartum cardiovascular disease that may itself require maternal intensive care [22], and of congenital heart defects in offspring [23]. Pre-gestational diabetes converges on the same dual outcome through a partly distinct pathway. In a meta-analysis of 81 studies comprising more than 137 million pregnancies, pre-gestational diabetes was associated with increased risk of hypertensive disorders of pregnancy, preterm delivery, and cesarean delivery. It was also associated with increased neonatal ICU admission, neonatal hypoglycemia, and perinatal mortality [24]. These observations are consistent with the cardio-obstetric concept of developmental programming, in which hypertensive disorders of pregnancy and maternal cardiometabolic risk factors such as diabetes affect maternal health and pregnancy outcome while also reducing fetal growth [25,26]. The association with absence of prenatal care is of a different character, plausibly reflecting undetected and unmanaged maternal disease rather than a shared biological mechanism. Despite this plausibility, these findings represent crude associations derived from aggregate birth certificate data. They may be influenced by confounding from correlated maternal characteristics, differences in clinical management, and underlying disease severity.

Marked racial disparities in concurrent maternal–neonatal ICU admission were observed, with nearly two-fold higher risk among American Indian or Alaska Native, Black, and Native Hawaiian or Other Pacific Islander mothers compared with White mothers. Unlike the clinical conditions discussed above, these gaps are unlikely to reflect inherent biological differences. They are instead consistent with evidence that disparities in severe maternal outcomes arise largely from structural inequities [27]. Socioeconomic disadvantage, reduced access to prenatal care, and a higher prevalence of comorbidities and pregnancy complications all contribute. These individual-level factors do not, however, fully explain the elevated rates observed among racial and ethnic minority women [27]. Differences in the quality and location of delivery care account for a further substantial share. In the United States, 75% of Black deliveries occur in a quarter of hospitals, and these hospitals have higher risk-adjusted severe maternal morbidity rates for both Black and White women [27]. Limited access to risk-appropriate obstetric care and timely referral to higher-level maternity services may contribute similarly to the disparities observed here. The elevated risks among pregnancies with no prenatal care, and to a lesser degree among WIC participants, further support the role of upstream social determinants. Economic disadvantage, barriers to healthcare access, and food insecurity frequently coexist and may contribute to delayed diagnosis and suboptimal management of chronic conditions during pregnancy. These unadjusted estimates cannot distinguish the independent contribution of race from correlated clinical, socioeconomic, and healthcare system factors. Birth certificate reporting of maternal morbidity has also been shown to differ by maternal race and ethnicity, with greater underreporting among Black and Hispanic patients [28,29]. Consequently, the disparities observed here may underestimate the true extent of these inequities, although this cannot be determined using aggregate surveillance data alone.

The relatively higher rate of maternal ICU admission without neonatal ICU admission at the earliest gestational ages is better understood as a competing-risk phenomenon than as evidence of disproportionately higher maternal critical illness. At the margins of viability, a substantial proportion of liveborn extremely preterm infants die shortly after birth. Because neonatal ICU admission can only occur among infants who survive long enough for intensive care to be initiated, early neonatal death acts as a competing event that precludes admission. This inflates the relative frequency of maternal ICU admission without neonatal ICU admission at very early gestational ages. Concurrent maternal–neonatal ICU admission at these gestations therefore captures a diminishing share of maternal–neonatal critical illness, not because neonatal illness is less severe, but because the neonatal component is precluded rather than absent. At later gestational ages, maternal ICU admission without neonatal ICU admission is more likely to reflect severe maternal illness in the setting of an infant who does not require intensive care. Some cases may nonetheless represent unrecorded term neonatal ICU admissions, since neonatal ICU admission is incompletely ascertained on birth certificates at term [30].

A central consideration in interpreting these findings is the validity of birth certificate data for identifying intensive care admission. Maternal and neonatal ICU admission in CDC WONDER are derived from birth certificate records, which incompletely ascertain both indicators. Maternal ICU admission has high specificity but low sensitivity and low positive predictive value relative to linked hospital discharge data [31], and is reported far less often than in national claims data [28]. Recorded maternal ICU admission is therefore affected by both false-negative and false-positive classification rather than under-ascertainment alone. Neonatal ICU admission is more reliably confirmed when recorded, but is also markedly incomplete. Fewer than half of medically documented admissions were captured in a multi-hospital validation against medical records [32]. Across 2.5 million linked deliveries, 38% of true admissions were missing, with more than half missed among term infants [30]. This is consistent with admissions occurring after certificate completion and with poor recording of transient neonatal ICU care [33]. Ascertainment also varies substantially across states and institutions. Sensitivity for neonatal ICU admission was high in one state but low in another and in New York City [32,34]. Hospital-level rates agreed poorly with a clinical registry across 103 California hospitals, with acceptable concordance in only one-third [33]. The absolute rates reported here are therefore likely to understate the true burden. More consequentially, under-ascertainment is non-random. Reporting completeness for maternal morbidity differs by maternal race and ethnicity, and this differential attenuates estimated disparities in birth certificate relative to claims data [28,29]. The subgroup associations reported here may therefore be biased rather than merely imprecise. Although available validation studies suggest attenuation, the magnitude of bias across all subgroup analyses cannot be determined from aggregate surveillance data. This concern is amplified for the concurrent phenotype. Because the outcome required both indicators, misclassification in each component compounds rather than averages out, and the sensitivity of the combined measure is lower than that of either alone. Beyond ascertainment, and as noted in the Methods, ICU admission reflects institutional practice, unit capacity, and referral patterns in addition to illness severity [13,14].

These findings have clinical and public health implications. Recognition of concurrent maternal–neonatal ICU admission as a distinct clinical phenotype highlights the need for care models that address maternal and neonatal risk together rather than in parallel. Most characteristics associated with this outcome are identifiable before critical illness develops, and some represent potentially modifiable clinical or healthcare factors. These associations are unadjusted, so the following implications should be read as hypotheses for evaluation rather than established intervention targets. The association with absence of prenatal care is the most difficult to address through prenatal services, since the pregnancies at highest risk are those not reached by them. It instead points to the importance of expanding coverage of preconception and prenatal services and reducing structural barriers to early care initiation. The associations with pre-pregnancy hypertension, pre-pregnancy diabetes, and hypertensive disorders of pregnancy are more amenable to intervention within existing care pathways. The observed associations align with current recommendations for routine screening for hypertensive disorders of pregnancy, gestational diabetes screening, and aspirin prophylaxis for women at increased risk of preeclampsia [35,36,37], supporting consistent implementation of evidence-based preventive care alongside optimization of pre-pregnancy cardiometabolic health. Early recognition may also facilitate referral of high-risk pregnancies to facilities with both obstetric adult ICU and neonatal ICU capability, as recommended for pregnant women requiring intensive care [13]. Multidisciplinary care involving obstetric, critical care, and neonatal teams is likewise recommended for the critically ill obstetric patient [13]. Such matching of risk to capability cannot be assumed. Deregionalization has meant that, in some areas, infants at high risk are cared for more broadly, including at smaller, lower-level units, with less favorable outcomes [38]. Neonatal intensive care resources at delivery hospitals for very preterm infants have also changed over the past decade [39], and regional neonatal ICU capacity shows little association with population-level perinatal risk [40]. Together, these observations reinforce the importance of integrating maternal and neonatal risk assessment into regional perinatal care planning. At the same time, because risk is patterned by structural as well as clinical factors, reducing this burden will likely require addressing determinants of access and care quality alongside individual clinical risk. The effectiveness of these measures for reducing concurrent maternal–neonatal ICU admission has not been established. This should be evaluated in prospective studies and linked maternal–neonatal datasets capable of assessing causal pathways and longer-term maternal and neonatal outcomes.

This study has several strengths, including the use of a large, nationally representative dataset covering more than 32 million singleton births, standardized data collection over a nine-year period, and sufficient statistical power to characterize a rare outcome and its temporal trends. The classification of births into four mutually exclusive phenotypes also permits maternal and neonatal critical care to be examined jointly rather than as separate outcomes. Several limitations nonetheless warrant consideration. The associations reported in Table 4 are unadjusted; because the analysis relied on aggregate tabulations, these estimates could not be mutually adjusted, and the independent contribution of any single characteristic cannot be separated from confounding by correlated maternal factors. Missingness for the stratifying covariates was generally low, ranging from below 0.1% to approximately 2.3% of births. Record-level missingness was associated with higher observed concurrent maternal–neonatal ICU admission rates and greater co-occurrence of unknown ICU indicators, whereas reporting-area noncollection was not. These patterns may reflect differences in documentation quality, underlying clinical complexity, or both. Therefore, the crude relative risks should be interpreted as descriptive associations among births with complete information. The restriction to singleton births, while necessary to avoid double counting of maternal ICU admission when neonatal outcomes are tabulated, means that the findings may not extend to multiple gestations, which carry higher maternal and neonatal risk. Finally, the aggregate structure of the data precludes causal inference, evaluation of individual-level causal pathways linking maternal and neonatal critical illness, and assessment of effect modification or mediation.

## 5. Conclusions

Concurrent maternal–neonatal ICU admission remained uncommon between 2016 and 2024 but increased more rapidly than either maternal or neonatal ICU admission alone. It was concentrated among pregnancies complicated by maternal comorbidities and earlier gestational age at delivery. These findings support interpreting concurrent maternal–neonatal ICU admission as a distinct clinical phenotype rather than viewing maternal and neonatal critical illness solely as separate outcomes. Because the estimates derive from aggregate birth certificate data, they describe documented concurrent maternal–neonatal ICU admission within the national vital statistics system rather than the true incidence of concurrent critical illness. Future studies should use patient-level or linked maternal–neonatal datasets. These should refine risk prediction, apply multivariable adjustment, investigate mediating mechanisms, evaluate preventive strategies, and assess longer-term maternal and neonatal outcomes.

## Figures and Tables

**Table 1:** Maternal and Neonatal Demographic and Clinical Characteristics (Singleton Births, 2024; *n* = 3,502,863).

Characteristics	*n* (%)
Maternal Race	
American Indian or Alaska Native	34,269 (1.0)
Asian	234,779 (6.7)
Black or African American	513,242 (14.7)
Native Hawaiian or Other Pacific Islander	13,986 (0.4)
White	2,600,851 (74.2)
More than one race	105,736 (3.0)
Age	
Mean (SD)	29.64 (5.82)
Under 15 years	1706 (0.05)
15–19 years	134,507 (3.8)
20–24 years	595,278 (17.0)
25–29 years	957,606 (27.3)
30–34 years	1,070,271 (30.6)
35–39 years	597,434 (17.1)
40–44 years	136,048 (3.9)
45–49 years	8991 (0.3)
50 years and over	1022 (0.03)
Timing of prenatal care initiation	
No prenatal care	83,073 (2.4)
First trimester	2,595,403 (74.1)
Second trimester	592,823 (16.9)
Third trimester	167,433 (4.8)
Unknown or Not Stated	64,131 (1.8)
Pre-pregnancy body mass index	
Mean (SD)	27.87 (6.88)
Underweight < 18.5	90,093 (2.6)
Normal 18.5–24.9	1,279,914 (36.5)
Overweight 25.0–29.9	958,160 (27.4)
Obesity I 30.0–34.9	586,909 (16.8)
Obesity II 35.0–39.9	293,447 (8.4)
Extreme Obesity III > 39.9	212,254 (6.1)
Unknown or Not Stated	82,086 (2.3)
Pre-pregnancy Diabetes	
Yes	45,775 (1.3)
No	3,453,815 (98.6)
Unknown or Not Stated	3273 (0.1)
Pre-pregnancy Hypertension	
Yes	115,662 (3.3)
No	3,383,928 (96.6)
Unknown or Not Stated	3273 (0.1)
Tobacco Use	
Yes	83,127 (2.4)
No	3,405,204 (97.2)
Unknown or Not Stated	14,532 (0.4)
Previous Cesarean Delivery	
Yes	537,153 (15.3)
No	2,962,437 (84.6)
Unknown or Not Stated	3273 (0.1)
Gestational age (Weeks)	
Mean (SD)	38.41 (1.95)
Under 20 weeks	997 (0.03)
20–27 weeks	17,241 (0.5)
28–31 weeks	25,114 (0.7)
32–35 weeks	133,410 (3.8)
36 weeks	129,785 (3.7)
37–39 weeks	2,402,447 (68.6)
40 weeks	621,722 (17.7)
41 weeks	161,286 (4.6)
42 weeks or more	9045 (0.3)
Unknown or Not Stated	1816 (0.1)
Birth Weight (g)	
Mean (SD)	3270 (558)
499 g or less	3926 (0.1)
500–999 g	13,940 (0.4)
1000–1499 g	19,281 (0.6)
1500–1999 g	42,609 (1.2)
2000–2499 g	165,200 (4.7)
2500–2999 g	678,499 (19.4)
3000–3499 g	1,401,335 (40.0)
3500–3999 g	920,354 (26.3)
4000–4499 g	223,348 (6.4)
4500–4999 g	29,036 (0.8)
5000–8165 g	3297 (0.1)
Unknown or Not Stated	2038 (0.1)

**Table 2:** Annual Numbers and Rates of Maternal ICU Admission, Neonatal ICU Admission, and Concurrent Maternal–Neonatal ICU Admission Among Singleton Births, United States, 2016–2024.

Year	Total Births	Maternal ICU Admission, No. (Rate; 95% CI)	Maternal ICU Admission Without Neonatal ICU Admission, No. (Rate; 95% CI)	Neonatal ICU Admission, No. (Rate; 95% CI)	Neonatal ICU Admission Without Maternal ICU Admission, No. (Rate; 95% CI)	Concurrent Maternal–Neonatal ICU Admission, No. (Rate; 95% CI)
2016	3,802,340	5663 (1.49; 1.45–1.53)	2994 (0.79; 0.76–0.82)	290,308 (76.35; 76.08–76.62)	287,639 (75.65; 75.38–75.91)	2669 (0.70; 0.68–0.73)
2017	3,717,152	5613 (1.51; 1.47–1.55)	2942 (0.79; 0.76–0.82)	293,015 (78.83; 78.55–79.10)	290,344 (78.11; 77.84–78.38)	2671 (0.72; 0.69–0.75)
2018	3,658,923	5687 (1.55; 1.51–1.60)	2939 (0.80; 0.77–0.83)	295,017 (80.63; 80.35–80.91)	292,269 (79.88; 79.60–80.16)	2748 (0.75; 0.72–0.78)
2019	3,618,297	5968 (1.65; 1.61–1.69)	3093 (0.85; 0.83–0.89)	297,892 (82.33; 82.05–82.61)	295,017 (81.53; 81.25–81.82)	2875 (0.79; 0.77–0.82)
2020	3,491,936	5488 (1.57; 1.53–1.61)	2648 (0.76; 0.73–0.79)	289,729 (82.97; 82.68–83.26)	286,889 (82.16; 81.87–82.45)	2840 (0.81; 0.78–0.84)
2021	3,537,532	6526 (1.84; 1.80–1.89)	2890 (0.82; 0.79–0.85)	301,845 (85.33; 85.04–85.62)	298,209 (84.30; 84.01–84.59)	3636 (1.03; 0.99–1.06)
2022	3,540,357	5963 (1.68; 1.64–1.73)	3101 (0.88; 0.85–0.91)	298,034 (84.18; 83.89–84.47)	295,172 (83.37; 83.09–83.66)	2862 (0.81; 0.78–0.84)
2023	3,472,364	5993 (1.73; 1.68–1.77)	3065 (0.88; 0.85–0.91)	302,674 (87.17; 86.87–87.46)	299,746 (86.32; 86.03–86.62)	2928 (0.84; 0.81–0.87)
2024	3,502,863	6386 (1.82; 1.78–1.87)	3151 (0.90; 0.87–0.93)	309,860 (88.46; 88.16–88.76)	306,625 (87.54; 87.24–87.83)	3235 (0.92; 0.89–0.96)

Rates are per 1000 singleton live births. Values in parentheses are the rate and 95% confidence interval, calculated using the Wilson score method for binomial proportions.

**Table 3:** Joinpoint Trend Analysis of Maternal ICU Admission, Neonatal ICU Admission, and Concurrent Maternal–Neonatal ICU Admission, United States, 2016–2024.

ICU Phenotype	Years	Selected Model (Joinpoints)	AAPC (95% CI)	*p*-Value
Maternal ICU admission	2016–2024	0	+2.48 (1.73–3.14)	<0.001
Maternal ICU admission without neonatal ICU admission	2016–2024	0	+1.66 (0.51–2.79)	0.005
Neonatal ICU admission	2016–2024	0	+1.65 (1.35–1.92)	<0.001
Neonatal ICU admission without maternal ICU admission	2016–2024	0	+1.64 (1.38–1.88)	<0.001
Concurrent maternal–neonatal ICU admission	2016–2024	0	+3.32 (1.67–4.88)	<0.001

Rates are per 1000 singleton live births. AAPC indicates average annual percent change. AAPCs were estimated using log-linear regression models. Because no joinpoints were identified, AAPC equals APC.

**Table 4:** Crude Risk of Concurrent Maternal–Neonatal ICU Admission According to Maternal Characteristics (Pooled U.S. Singleton Births, 2016–2024; N = 32,341,764).

Characteristics	No./Total	Frequency, No./1000 Live Births (95% CI)	RR (95% CI)
Maternal Race (*n* = 32,341,764)			
American Indian or Alaska Native	380/313,912	1.21 (1.09–1.33)	1.75 (1.58–1.93)
Asian	1748/2,147,811	0.81 (0.78–0.85)	1.17 (1.12–1.23)
Black or African American	6922/5,052,677	1.37 (1.34–1.40)	1.98 (1.92–2.03)
More than one race	736/892,120	0.83 (0.77–0.88)	1.19 (1.11–1.28)
Native Hawaiian or Other Pacific Islander	169/114,209	1.48 (1.26–1.70)	2.14 (1.84–2.48)
White	16,509/23,821,035	0.69 (0.68–0.70)	1
Maternal age group (*n* = 32,341,764)			
<20 years	1147/1,471,987	0.78 (0.73–0.82)	1.23 (1.15–1.32)
20–24 years	3804/6,021,234	0.63 (0.61–0.65)	1
25–29 years	6066/9,177,764	0.66 (0.64–0.68)	1.05 (1.00–1.09)
30–34 years	7651/9,514,058	0.80 (0.79–0.82)	1.27 (1.22–1.32)
35–39 years	5698/5,002,987	1.14 (1.11–1.17)	1.80 (1.73–1.88)
40–44 years	1899/1,075,417	1.77 (1.69–1.85)	2.80 (2.65–2.95)
≥45 years	199/78,317	2.54 (2.19–2.89)	4.02 (3.49–4.64)
Trimester Prenatal Care Began (*n* = 31,627,499)			
First trimester	16,548/24,376,620	0.68 (0.67–0.69)	1
Second trimester	4451/5,183,968	0.86 (0.83–0.88)	1.26 (1.22–1.31)
Third trimester	1065/1,445,037	0.74 (0.69–0.78)	1.09 (1.02–1.16)
No prenatal care	2581/621,874	4.15 (3.99–4.31)	6.11 (5.87–6.37)
WIC participation (*n* = 31,980,520)			
No	16,278/21,157,524	0.77 (0.76–0.78)	1
Yes	9134/10,822,996	0.84 (0.83–0.86)	1.10 (1.07–1.13)
Pre-pregnancy BMI category (*n* = 31,614,254)			
Underweight < 18.5	704/944,923	0.75 (0.69–0.80)	1.21 (1.12–1.30)
Normal 18.5–24.9	7920/12,811,078	0.62 (0.60–0.63)	1
Overweight 25.0–29.9	5995/8,558,158	0.70 (0.68–0.72)	1.13 (1.10–1.17)
Obesity I 30.0–34.9	4288/5,011,532	0.86 (0.83–0.88)	1.38 (1.33–1.44)
Obesity II 35.0–39.9	2484/2,494,624	1.00 (0.96–1.03)	1.61 (1.54–1.68)
Extreme Obesity III > 39.9	2710/1,793,939	1.51 (1.45–1.57)	2.44 (2.34–2.55)
Pre-pregnancy diabetes (*n* = 32,318,090)			
No	24,633/31,977,602	0.77 (0.76–0.78)	1
Yes	1723/340,488	5.06 (4.82–5.30)	6.57 (6.26–6.90)
Gestational diabetes (*n* = 32,318,090)			
No	23,703/29,949,720	0.79 (0.78–0.80)	1
Yes	2653/2,368,370	1.12 (1.08–1.16)	1.42 (1.36–1.47)
Pre-pregnancy hypertension (*n* = 32,318,090)			
No	23,365/31,525,517	0.74 (0.73–0.75)	1
Yes	2991/792,573	3.77 (3.64–3.91)	5.09 (4.90–5.29)
Gestational hypertension (*n* = 32,318,090)			
No	20,305/29,710,265	0.68 (0.67–0.69)	1
Yes	6051/2,607,825	2.32 (2.26–2.38)	3.40 (3.30–3.49)
Eclampsia (*n* = 31,677,260)			
No	24,444/31,596,635	0.77 (0.76–0.78)	1
Yes	1444/80,625	17.91 (16.99–18.83)	23.15 (21.96–24.40)
Tobacco use during pregnancy (*n* = 32,202,343)			
No	23,380/30,550,978	0.77 (0.76–0.78)	1
Yes	2524/1,651,365	1.53 (1.47–1.59)	2.00 (1.92–2.08)
Infections during pregnancy (*n* = 32,256,609)			
None checked	24,597/31,380,006	0.78 (0.77–0.79)	1
At least one checked	1496/876,603	1.71 (1.62–1.79)	2.18 (2.07–2.29)

Reference categories are indicated by RR = 1. Denominators vary by characteristic because missing or unknown responses for that characteristic were excluded (ranging from below 0.1% to approximately 2.3% across characteristics).

## Data Availability

The data supporting the findings of this study are publicly available from the Centers for Disease Control and Prevention (CDC) Wide-ranging Online Data for Epidemiologic Research (CDC WONDER) Natality database at https://wonder.cdc.gov/natality.html (accessed on 19 June 2025). The data analyzed during the current study can be accessed through the CDC WONDER online query system, subject to the database terms of use. No new datasets were generated during this study.

## Supplementary Materials

The following supporting information can be downloaded at: https://www.mdpi.com/article/10.3390/jcm15166357/s1, Table S1: Concurrent maternal–neonatal ICU admission and ICU-indicator completeness according to missingness in selected maternal characteristics, US singleton live births, 2016–2024.

## Abbreviations

The following abbreviations are used in this manuscript:

AAPC	Average annual percent change
APC	Annual percent change
CDC	Centers for Disease Control and Prevention
ICU	Intensive Care Unit
WIC	Women, Infants, and Children Program
WONDER	Wide-ranging Online Data for Epidemiologic Research
