# Supplementary material for: The Overlap of Maternal and Neonatal Critical Care Admission in the United States: Trends and Risk Factors in 2016–2024
Source: J Clin Med. 2026 Aug 18;15(16):6357. doi: 10.3390/jcm15166357 (PMC13513881; doi:10.3390/jcm15166357)
Supplement: Supplementary file 1 [file jcm-15-06357-s001.zip › jcm-4418456-supplementary.pdf]

Table S1. Concurrent maternal–neonatal ICU admission and ICU-indicator completeness according to missingness in selected maternal characteristics, US singleton live births, 2016–2024.

Characteristic	Availability category	Births with known ICU status	Concurrent ICU admissions	Rate per 1,000	Births with ≥1 ICU indicator unknown	% with ≥1 ICU indicator unknown
**Pre-pregnancy BMI**	Recorded	31,614,254	24,101	0.76	63,019	0.20
	Unknown or not stated	727,510	2,363	3.25	13,271	1.79
**Trimester prenatal care began**	Recorded	31,627,499	24,645	0.78	61,377	0.19
	Unknown or not stated	714,265	1,819	2.55	14,872	2.04
**WIC participation**	Recorded	31,980,520	25,412	0.79	65,887	0.21
	Unknown or not stated	361,244	1,052	2.91	10,412	2.80
**Eclampsia**	Recorded	31,677,260	25,888	0.82	58,139	0.18
	Unknown or not stated	23,627	108	4.57	17,567	42.64
	Not reported by jurisdiction	640,877	468	0.73	589	0.09

BMI, body mass index; ICU, intensive care unit; WIC, Special Supplemental Nutrition Program for Women, Infants, and Children.

Rates are per 1,000 singleton live births with known maternal and neonatal ICU admission status. "Recorded" includes all non-missing response categories for the characteristic. "Not reported by jurisdiction" indicates reporting areas that did not collect the variable (for eclampsia: South Carolina, 2016–2021; Tennessee, 2016–2019). Percentages with ≥1 ICU indicator unknown were calculated using all births within each availability category, including those excluded from the analytic cohort because maternal or neonatal ICU admission status was unknown. CDC WONDER suppresses cells containing fewer than 10 births; therefore, counts involving unknown ICU indicators are slight underestimates.
